# Triple-core-hole states produced in the interaction of solid-state density plasmas with a relativistic femtosecond optical laser

**DOI:** 10.1038/s41598-018-29484-6

**Published:** 2018-07-23

**Authors:** Cheng Gao, Yongjun Li, Pengfei Liu, Xiaohui Fan, Jiaolong Zeng

**Affiliations:** Department of Physics, College of Liberal Arts and Sciences, National University of Defense Technology, Changsha, 410073 Hunan P. R. China

## Abstract

Extremely exotic dense matter states can be produced in the interaction of a relativistic femtosecond optical laser with a solid density matter. Here we theoretically investigate triple-core-hole (TCH) states produced by an intense polychromatic x-ray field formed by hot electrons in the interaction of a relativistic femtosecond optical laser with a thin silver foil. X-ray emission spectra of solid-density silver plasmas show unambiguously the production of TCH states at an electron temperature of a few hundreds of eV and radiative temperature of 1–3 keV of the polychromatic x-ray field. Practical calculations show that the emissivity originating from the TCH states exceeds that from the single- and double-core-hole states in Ne-like Ag^37+^ at electron temperature of ~500 eV and radiative temperature of ~1500 eV. For the neighbouring ionization stages of Ag^36+^ and Ag^38+^, TCH emissivity is roughly equivalent or comparable to that from the single- and double-core-hole states. Present work deepens our insight into investigation of the properties of extremely exotic states, which is important in high energy density physics, astrophysics and laser physics.

## Introduction

Atoms with inner-shell vacancies such as single- and double-core-hole (SCH/DCH) states have been produced and observed by different methods such as collisions with highly charged ions and electron beams^1–4^, interaction with synchrotron radiation^5–7^ and laser beams such as ultra-intense ultrafast x-ray free electron laser (XFEL)^8–13^ and intense optical lasers^14–20^. These exotic atomic states contain rich information of the surrounding environment and can be helpful in many research fields such as chemical analysis^21–23^, x-ray atomic laser^24,25^ and warm and hot dense matter^26–29^.

Although many researches on SCH and DCH states have been carried out in the past decades, however, very few studies are reported in the literature on the production of triple-core-hole (TCH) states^30–32^. Wallis *et al*.^30^ theoretically studied the formation of TCH states in the 2p shell of an argon atom interacting with a free electron laser pulse. Carravetta *et al*.^31^ calculated the energies of TCH states of molecular nitrogen. These studies deepened our understanding for TCH states. However, the fraction of TCH states in these investigations is very low. To the best of our knowledge, no work is reported on the production and decay properties of TCH states in hot dense plasmas. In the previous researches^1–20^, SCH and DCH states were produced by the ionization of low-Z elements which have one or two K-shell vacancies. To experimentally produce TCH states, one better utilizes medium- or high-Z materials, which have fully occupied inner L-shell and M-shell electrons. Three L-shell electrons can be ionized or excited and hence TCH states are produced. The investigation of TCH states gives rise to a great challenge both experimentally and theoretically. Experimentally, TCH states are highly excited and decay very fast, and thus ultra-intense ultrafast laser pulses are required. For high-Z materials, the greatly increased re-absorption compared with low-Z materials prevents accurate measurement of the spectra^33^. Theoretically, myriad microscopic atomic processes including the excitation and ionization initiated by electrons and photons take part in the ionization balance of many ionization stages in high-Z plasmas. The determination of the population distributions including the TCH states represents a great challenge because so many quantum states are involved. For the exotic TCH states, there are a large number of atomic states and their energies are in general highly degenerate, which gives rise to great difficulty in obtaining accurate atomic data^34^. In order to obtain spectroscopic accuracy in theory, one has to employ a detailed level accounting method to obtain the correct line intensity and position in the spectral modeling^35,36^. This could further increase the difficulty of high-Z plasma spectral simulation.

In this work, radiative properties of TCH states are investigated for solid-density silver plasmas produced in the interaction of a relativistic femtosecond optical laser. Atomic kinetic calculation by using a detailed level accounting method showed that the emissivity of TCH states exceeds that of SCH and DCH states for Ne-like Ag^37+^ and is comparable for the nearby Na-like Ag^36+^ and F-like Ag^38+^ ionization stages at electron temperature of 500 eV and radiative temperature of 1500 eV. Intense x-ray radiation field is produced by fast electrons refluxing in the interaction of a relativistic optical laser with a thin silver foil^16,19^. Multiple-core-hole state emission properties of Ag are investigated systematically at electron temperature of 30–1000 eV and radiative temperature of 1–3 keV. The optimum conditions to produce TCH states are investigated systematically.

## Results

**Figure 1:**
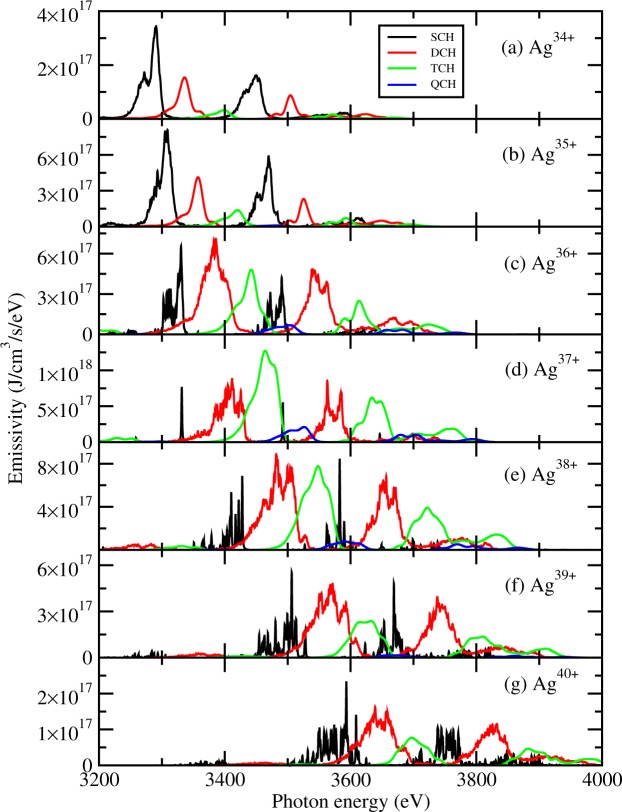
Emissivity of single and multiple-core-hole states of different ionization stages in a solid-density silver plasma at an electron temperature of Te = 500 eV and radiative temperature of Tr = 1500 eV.

**Figure 2:**
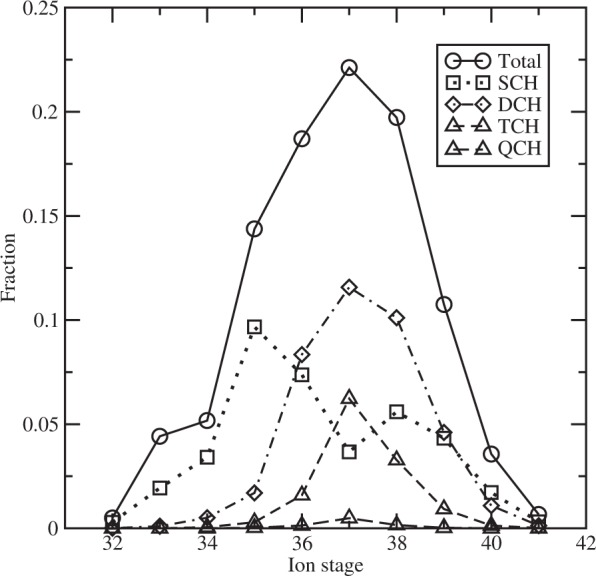
Charge state distribution of solid-density Ag plasma at an electron temperature of 500 eV and radiative temperature of 1500 eV. The fractions of populations are determined to be 4.4%, 5.2%, 14.4%, 18.7%, 22.1%, 19.7%, 10.8%, and 3.6%, respectively, for Ag^33+^-Ag^40+^. Relative population distributions of SCH, DCH, TCH and QCH states are also given. A fraction of 1% hot electrons with a temperature of 10 keV is included in the calculation.

In Fig. 1 we show the emissivity contributed by the multiple-core-hole states of different ionization stages in a solid-density silver plasma at an electron temperature of 500 eV and a Planck radiation field with a temperature of 1500 eV. The charge state distribution and emissivity of plasmas are investigated by solving a rate equation which connects the involved quantum states^37^. A fraction of 1% hot electrons with a temperature of 10 keV is included in the calculation and the thickness of the silver foil is 0.5 *μm*. At the given plasma condition, the charge state distribution is shown in Fig. 2 for the dominant ionization stages of Ag^33+^-Ag^40+^. Ag^37+^ has a highest population fraction of TCH states among all ionization stages, which accounts for 6.3%. The population fraction of SCH and DCH states of Ag^37+^ is 3.7% and 11.6%, respectively. The TCH states originate from 1s^2^(L)^−3^ nln’l’n”l” (n,n’,n” ≥3), for examples 1s^2^2s^2^2p^3^3s^2^3p, 1s^2^2s^2^2p^3^3s^2^3d, 1s^2^2s2p^4^3s^2^3p, 1s^2^2s2p^4^3s^2^3d, 1s^2^2s^0^2p^5^3s^2^3p, 1s^2^2s^0^2p^5^3s^2^3d, etc. The ionization stages with the next highest TCH population fraction are Ag^38+^ and Ag^36+^ which account for 3.3% and 1.6%, respectively. The corresponding population fractions of SCH and DCH states are 5.6% and 10.1% for Ag^38+^, and 7.4% and 8.3% for Ag^36+^, respectively.

The emission lines shown in Fig. 1 originate dominantly from *3d-2p* and *3p-2s* bound-bound transitions. Contributions from higher transition arrays such as *4d-2p* are much smaller. In this photon energy region, the continuous emissions from the free-bound and free-free processes are weaker by at least two orders of magnitude. The emission lines from a particular transition array are obviously separated into two groups due to the relativistic orbital splitting^38^. The emissivity contributed by the TCH states is surprisingly larger than the summation of SCH and DCH states for Ne-like Ag^37+^. For this ion, SCH states emit only two narrow lines, whereas DCH and TCH states provide a wide quasi-continuum emission with a range of photon energy of more than 100 eV. Intensity of TCH emission is comparable to that of SCH and DCH emission for Na-like Ag^36+^ and F-like Ag^38+^. We can also observe the production of quadruple-core-hole (QCH) states in the interaction of laser with the silver material although the population of the QCH states is much smaller than TCH states.

The only possible origin for the production of TCH and QCH states is the intense polychromatic x-ray field which is formed by hot electrons produced in the interaction with the ultra-intense laser^16–19^. The first ionization potential of Ag^37+^ is 5558 eV^39^, which is much higher than the thermal energy at the given electron temperature (500 eV). As a result, these free electrons cannot effectively ionize the material to such a high ionization stage of Ag^37+^. Hot electrons have enough energy and can indeed ionize the atoms and ions in the plasma, yet the ionization efficiency of the inner-shell electrons of 2p and 2s is much smaller than the x-ray radiative field^18^. It is found that TCH states can be effectively produced in a Planck radiation field with a temperature of 1500 eV at the given electron temperature of 500 eV. Such a radiation field has a largest intensity (and photon population) at a photon energy of 4230 eV, which can effectively photo-excite 2p electrons to 3d orbital and 2s electrons to 3p orbital with an excitation energy of about 3500 eV and 3700 eV for Ag^37+^.

**Figure 3:**
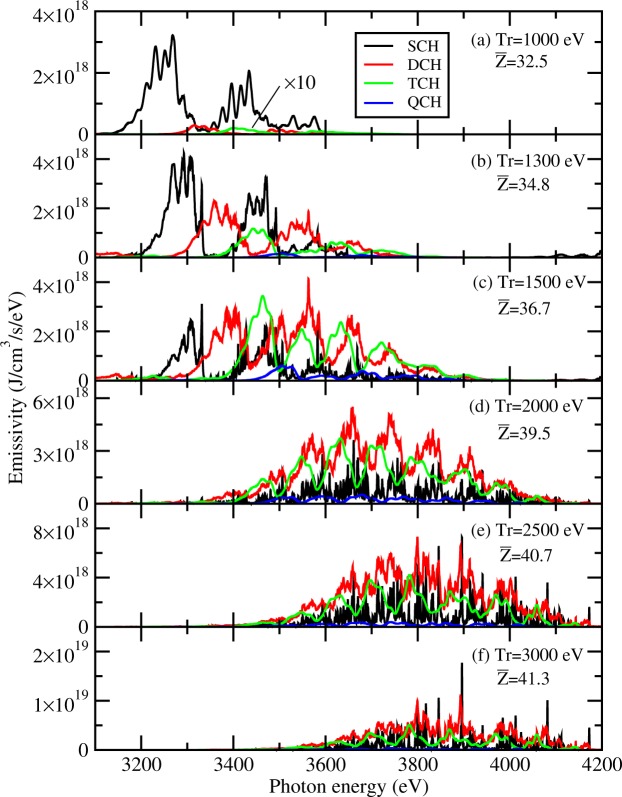
Emissivity of single and multiple-core-hole states of solid-density Ag plasmas at an electron temperature of Te = 500 eV and different radiative temperature of Tr = (**a**) 1000 eV, (**b**) 1300 eV, (**c**) 1500 eV, (**d**) 2000 eV, (**e**) 2500 eV and (**f**) 3000 eV, respectively. Emissivity of TCH states in panel (a) is multiplied by a factor of 10 for better visibility. Average ionization degree is given in each panel.

The efficiency of producing TCH states with radiation field of different temperature can be further seen from Fig. 3, which shows the emissivity of the plasmas produced in the interaction of laser with silver foils. The emissivity contributed by the SCH, DCH, TCH, and QCH states is given separately to evaluate their respective contributions. Here we fix the electron temperature of Ag plasmas to be 500 eV for all considered cases. At the radiative temperature of 1000 eV, only a small fraction (0.9%) of DCH states can be produced and most of the production belongs to SCH states. The fraction of DCH and TCH states increases very fast with the increase of radiation temperature. At the radiative temperature of 1500 eV, TCH and DCH emissions exceed those of SCH. In addition to TCH, QCH states begin to appear in the plasmas. With the further increasing of the radiative temperature to 2000 eV, DCH states contribute the largest emissivity and TCH emission is stronger than the SCH emission. Therefore we can conclude that the favorable condition to produce the TCH states is a radiation field with a temperature of ~1500 eV for Ag plasmas at an electron temperature of 500 eV.

**Figure 4:**
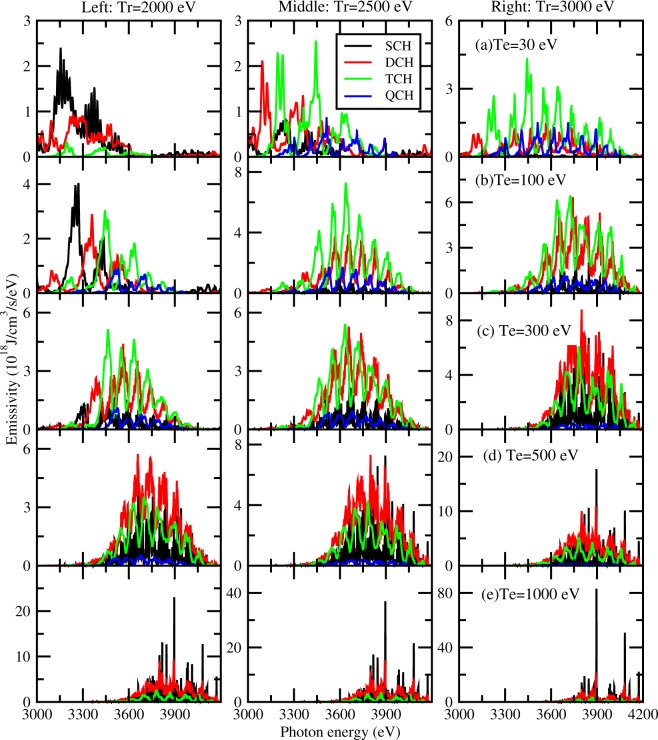
Emissivity of single and multiple-core-hole states of solid-density Ag plasmas at radiative temperatures of Tr = 2000 eV (left), 2500 eV (middle) and 3000 eV (right). The electron temperature is set to be 30 eV, 100 eV, 300 eV, 500 eV and 1000 eV, respectively, from top to bottom.

The production of TCH states is also closely related with the electron temperature of the plasmas. In Fig. 4, we show the emissivity of plasmas at different electron temperature from 30 eV to 1000 eV. The radiation field temperature is assumed to be 2000 eV (left), 2500 eV (middle) and 3000 eV (right), respectively. For a particular temperature of radiation field, we can see that the production of TCH states is sensitive to electron temperature. First let us look at the case of radiative temperature of 2000 eV. At the lower electron temperature (30 eV), emissivity of SCH states dominates and TCH emissivity is much weaker. With the electron temperature increasing to 300 eV, TCH emissions are the most important part and even QCH emissions appear. As the electron temperature further increases, contribution of TCH emission decreases. At such a radiative temperature, the favorable electron temperature to produce TCH states is around 300 eV. Similar characteristics of TCH emission could be found for the cases of radiative temperature of 2500 eV and 3000 eV. The optimized electron temperature to produce TCH states decreases as the radiative temperature increases, which is around 100 eV and 30 eV at the radiative temperature of 2500 eV and 3000 eV, respectively. By utilizing these features of emission spectra, we can deduce the plasma condition produced in the interaction with ultra-intense lasers.

## Discussion

In what follows we explain the underlying physical processes which determine the charge state distribution and emissivity of Ag plasmas. From the inspection of Fig. 4, we find that the emissivity contributed by TCH states at the electron temperature of 1000 eV is always small regardless of the radiative temperature. This is because the high electron temperature makes the Ag plasma in a condition of highly ionized state. The electrons in the plasma effectively ionize the matter into the ionization stages around Ag^37+^. The introduction of radiation field further ionizes the matter into a higher ionized state than Ag^37+^. As a result, the fraction of TCH states is negligibly small. With the decrease of electron temperature, the plasma electrons can no longer ionize the matter into a highly charged state and therefore the radiation field plays a more important role in producing the TCH states. The radiation field with a temperature given in this work can effectively ionize or excite the 2s and 2p electrons, yet the photons do not have enough energy to ionize the 1s electrons. On the other hand, the rate of photoionization of outer-shell electrons such as 3s and 3p is much smaller. This is a favorable situation to produce multiple-core-hole states in the plasma, in particular for Ag^37+^. For this ion, it has a closed atomic structure 1s^2^2s^2^2p^6^ and hence the singly excited states 1s^2^2s^2^2p^5^ nl are bound ones with a relatively long natural lifetime. However, the multiple-core-hole states of charge states below Ag^37+^ have a shorter natural lifetime because they decay via Auger as well as radiative processes, unlike the excited states 1s^2^2s^2^2p^5^ nl of Ag^37+^ via radiative decay only. As a result, there is an optimized electron temperature to produce TCH states at a given radiative temperature.

**Figure 5:**
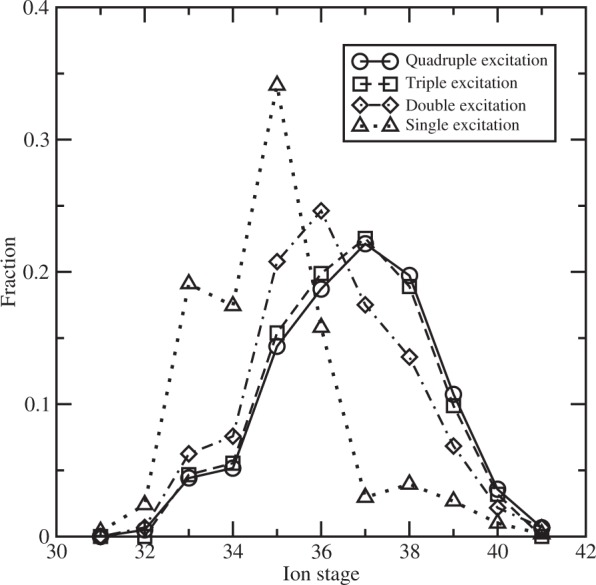
Convergence of charge state distribution of Ag plasma with scale of atomic configurations at an electron temperature of 500 eV and radiative temperature of 1500 eV. Results of quadruple, triple, double and single excitations represent the calculations including multiple-core-hole states up to QCH, TCH, DCH and SCH states, respectively.

The present work utilizes a large-scale rate equation approach to determine the level populations and thus the convergence of our results can be guaranteed^40^. In all our calculations, we have included ionization stages from Ag^21+^ to the bare ion. For each ionization stage, in addition to the valence electron excitation states, we have further included the quantum states of singly, doubly, triply and quadruply excited configurations from the L shells (2s and 2p) to ensure the completeness of the atomic model. Take the Ne-like Ag^37+^ as an example to illustrate the scale of included quantum states. The quantum states belonging to the following electronic configurations have been included in our calculations: (1)^2^(2)^8^, (1)^2^(2)^7^ nl, (1)^2^(2)^6^ nln’l’, (1)^2^(2)^5^(3)^3^, (1)^2^(2)^5^(3)^2^ nl, (1)^2^(2)^4^(3)^4^ and (1)^2^(2)^4^(3)^3^ nl. Here the notation (N)^M^ means M electrons are occupied in the orbital shell with principle quantum number N and therefore the designation (2)^4^ in the configurations means four electrons have been excited from the orbital of 2s and 2p. For lower charge states than Ag^37+^, many more configurations originating from the valence electron excitation have also been included. The maximal principal quantum numbers n and n’ are determined by the plasma condition. For the solid-density Ag plasma at the electron temperature of 500 eV, it is calculated to be 5 for Ag^37+^ and 6 for Ag^39+^ due to the ionization potential depression. The convergence trend can be seen from Fig. 5, which shows the results with different scale of excitation. Nearly converged results have been obtained for including the triple excitation.

The ionization potential depression is significant for the solid-density Ag plasma which makes the excited states with a principal quantum number larger than 5 being merged into the continuum for Ag^37+^. In this work, we have considered the effects of screening potential caused by the plasma environment on atomic structure and atomic processes. Briefly, the plasma screening potential originates from the interaction with surrounding free electrons and ions in the plasma, especially free electrons. In this work, we used the screening potential based on the average atom model^41^. It is determined by the plasma micro-field of the free electrons, which have a Thomas-Fermi distribution. The screening potential is added to the self-consistent potential of isolated ion and therefore influences the energy levels, ionization energy and spectral line width^42^. The plasma screening has a more pronounced effect for the energy level shift and ionization potential, yet the effect is much smaller for the transition energy and probability. A detailed quantitative demonstration and discussion are beyond the scope of the present work.

In summary, we investigated exotic atomic TCH states production in the interaction of a relativistic optical laser with a thin silver foil. We predicted that TCH emissivity exceeds SCH and DCH emissivity for Ag^37+^ and is comparable for Ag^36+^ and Ag^38+^ at electron temperature of 500 eV and radiative temperature of 1500 eV. The optimized electron temperature to produce TCH states decreases with radiative temperature increasing, which is around 300 eV, 100 eV and 30 eV, respectively, for the corresponding radiative temperature of 2000 eV, 2500 eV and 3000 eV. The intense x-ray radiation is generated by fast electrons which are accelerated in ultra-intense relativistic optical laser in the interaction with a thin silver foil. We estimated the femtosecond optical laser intensity should be about 10^21^ W/cm^2^ according to previous investigations on aluminium and silicon^16–19^. Extremely exotic atomic states with four or even more core-holes could be produced by irradiating ultra-intense ultrafast optical lasers on thin high-Z element foils. Our results should be useful to understand and interpret related experiments.

## Methods

### Rate equation

In a non-local thermodynamic equilibrium (NLTE) plasma, the population distribution of different quantum states is determined by the relevant microscopic atomic processes. The population *n*_*i*_ of the state *i* can be obtained by solving a rate equation^43,44^

$$\frac{d{n}_{i}}{dt}=\sum _{j}{n}_{j}{R}_{ji}-{n}_{i}\sum _{j}{R}_{ij}$$

where *R*_*ji*_ and *R*_*ij*_ represent the populating rate from state *j* to *i* and the de-populating rate from state *i* to *j*, respectively. In the calculations, the following microscopic atomic processes are included, i.e., photoexcitation, photoionization, electron impact excitation, electron impact ionization and autoionization, as well as their inverse processes. In this work, the rate equation is solved at the steady-state assumption where the left hand of the rate equation equals zero. The opacity effect is treated by an escape factor approximation^45,46^.

We utilized a hybrid method to obtain high precision x-ray emission spectra. Firstly, a detailed relativistic configuration accounting model is applied in the rate equation to obtain the population distributions in the relativistic configurations. Then the populations of the fine-structure levels belonging to each particular relativistic configuration are determined by assuming the fine-structure levels are at equilibrium in the relativistic configurations which are belonging to. The fine-structure level populations are obtained by the formula

$${N}_{l}={n}_{C}\frac{{g}_{l}{e}^{-{E}_{l}/kT}}{\sum _{l\in C}{g}_{l}{e}^{-{E}_{l}/kT}},$$

where *g*_*l*_ and *E*_*l*_ are the statistical weight and energy of the fine-structure level *l* belonging to the relativistic configuration *C*, *N*_*l*_ and *n*_*c*_ are the populations of the fine-structure level *l* and the relativistic configuration *C*, respectively. Finally, the emission properties are obtained by using the radiative transition data in the level-level formalism and the population distribution of the fine-structure levels.

### Atomic code DLAYZ used for modeling

We use code DLAYZ to perform the calculation^43^, which is a versatile code for investigating population kinetics and radiative properties of NLTE plasmas. The complete set of atomic data including radiative transition probability, microscopic cross section of photoionization, electron impact excitation and electron impact ionization, and autoionization rate are obtained by using the Flexible Atomic Code (FAC)^47^. In FAC, atomic orbital wave functions are obtained by solving Dirac-Fock equation which is used to construct the configuration state wave function. The atomic state wave functions are expanded by the configuration state wave function with the same parity and angular momentum^47^. The continuum wave functions are obtained based on the relativistic distorted wave methods. The basic atomic data can be calculated after the atomic state wave functions are obtained.
